# Effect of zinc supplementation as an adjuvant to corticosteroid treatment in patients with oral lichen planus: A systematic review

**DOI:** 10.34172/japid.2023.017

**Published:** 2023-09-30

**Authors:** Ayla Bahramian, Mona Rahbaran, Maria Bahramian, Sepideh Bohlouli, Katayoun Katebi

**Affiliations:** ^1^Department of Oral Medicine, Faculty of Dentistry, Tabriz University of Medical Sciences, Tabriz, Iran; ^2^Faculty of Dentistry, Tabriz University of Medical Sciences, Tabriz, Iran; ^3^Department of Anesthesiology and Intensive Care Medicine, Faculty of Medicine, Tabriz University of Medical Sciences, Tabriz, Iran

**Keywords:** Adrenal cortex hormones, Gingivitis, Lichen planus, Oral, Systematic review, Zinc

## Abstract

**Background.:**

Oral lichen planus (OLP) and one of its main presentations, desquamative gingivitis, are common diseases with no definite treatment. Zinc deficiency has a critical role in the pathogenesis of oral mucosal diseases. The current study systematically reviewed the effect of zinc in addition to topical corticosteroids in the treatment of OLP.

**Methods.:**

English articles in PubMed, Web of Sciences, Embase, and Scopus were searched until August 2022. The differences in symptoms were analyzed, including pain, burning sensation, and lesion sizes in patients with lichen planus receiving zinc supplementation as an adjuvant to corticosteroid treatment.

**Results.:**

A total of 148 articles related to the searched keywords were found. Eventually, two clinical trials were selected. The total population of studied individuals included 60 patients. Due to the high heterogeneity between the studies, meta-analysis was not possible. Administering zinc, in addition to corticosteroids, did not improve the symptoms compared to corticosteroid monotherapy.

**Conclusion.:**

Considering the limited number of studies and lack of sufficient evidence, it is not currently possible to reach a definite conclusion regarding the effects of zinc on OLP.

## Introduction

 Oral lichen planus (OLP) is an autoimmune disease mediated by T cells. CD8^+^ T cells play an active role in lesion formation by secreting various cytokines, including interleukin-12 and tumor necrosis factor-α. The condition disrupts the integrity of the basal lamina.^1,2^ In terms of clinical manifestations, this disease appears as lesions with white striae, papules, erosions, ulcers, and plaques that affect the buccal mucosa, gingiva, and tongue.^3-5^ The symptoms of this disease include surface roughness of oral mucosa, the sensitivity of oral mucosa to warm or spicy foods, painful oral mucosa, white or red spots on the oral mucosa, or oral ulcers.^6,7^ Desquamative gingivitis is one of the primary and common presentations of OLP.^8,9^ Desquamative gingivitis is a non-plaque-induced lesion of the gingiva. It clinically presents as erythema, erosion, and ulceration of the gingiva and oral mucosa. Most of the desquamative gingivitis lesions are manifestations of OLP.^10^ Since this disease causes severe patient discomfort, proper treatment is essential.

 The medical treatments of OLP mainly aim to relieve the symptoms and prevent future recurrences. Topical corticosteroid administration is the primary choice of treatment. However, this method is associated with various side effects, including dry mouth, thinning of the oral mucosa, and *Candida albicans* overgrowth in the oral cavity.^11-14^ Considering the mentioned complications, the application of various adjuvant treatments, including the administration of immunomodulators, immunosuppressants, retinoids, cytotoxic and antibacterial agents, and alternative therapies, including lycopene, curcumin, and zinc, are among the suggested treatments.^15-19^

 Zinc, an essential micronutrient, has more than 300 metalloenzymes required for gene transcription and fat, protein, and nucleic acid regulation. Zinc plays a vital role in maintaining the immune system’s function and wound healing by regulating ribonuclease, DNA-RNA polymerases, and thymidine kinase.^20^ It also regulates the performance of macrophages, neutrophils, and natural killer cells.^21^ Zinc has an anti-inflammatory effect by inhibiting cytokines and can induce apoptosis. The reduction of zinc level is linked to inhibiting the oxidant‒antioxidant system.^22-24^ On the other hand, zinc is an antioxidant that can stabilize the cell membrane, playing a part in the pathogenesis of OLP.^25,26^

 A study by Gholizadeh et al^27^ revealed reduced zinc serum levels in OLP patients, indicating zinc’s promising role in preventing and treating OLP. A study by Bao et al^28^ indicated that zinc deficiency might be associated with the pathogenesis of common lesions of the oral mucosa. Zinc supplementation might be a beneficial therapy for oral mucosa diseases, including OLP.

 Considering the role of corticosteroids as the primary treatment for OLP and the suggested adjuvant role of zinc in increasing the corticosteroids’ therapeutic effects, the present systematic review investigated the effect of zinc supplementation in addition to the topical administration of corticosteroids on OLP.

## Methods

 The present study was registered in PROSPERO (ID: CRD42022366032).

 The reporting method of the present systematic review conformed to PRISMA guidelines,^29^ and its research question was based on patient/population, intervention, comparison, and outcomes (PICO). The main question of the study was: “Does zinc supplementation as an adjuvant to corticosteroid (I) treatment in patients with oral lichen planus (P) decrease pain and burning sensation (O) compared to corticosteroid therapy alone (C)?”

 This systematic review investigated the articles published in English in Scopus, Web of Sciences, PubMed, and Embase databases until April 2023. MeSH and free keywords with ‘OR and AND’ operators and their combinations were used for the search. The keywords included oral lichen planus, lichenoid reaction, lichen planus, OLP, corticosteroid, zinc, triamcinolone, fluocinolone, betamethasone, dexamethasone, adrenal cortex hormones, corticoids, steroids, Adcortyl, burning sensation, visual analog scale, pain, burning pain, glossalgia, and Thongprasom scale.

 Two experts conducted the screening process after extracting the articles from the databases. In the first stage, two independent reviewers investigated the titles and abstracts of the studies according to the inclusion and exclusion criteria. The third reviewer was consulted to resolve the disagreements. In the next step, the full texts of the selected articles were studied.

 The revised Cochrane risk-of-bias tool for randomized trials (RoB2) was used to evaluate the risk of bias in the selected articles.^30^

 Microsoft Excel was used to organize the extracted data of each study. The extracted data included the first author’s name, publication year, sample size, medication dosage, and study results, including pain levels and lesion sizes.

 The inquiries were limited to human studies. The exclusion criteria were case reports, review studies, animal studies, experimental studies without control groups, and lack of access to the full text of the article. The study outcomes included improvements in pain and burning symptoms based on visual analog scale (VAS) and changes in the size of the lesions in lichen planus patients.

 Because of the high heterogeneity between the studies, meta-analysis was not possible, and the results of each study were reported separately.

## Results

**Figure 1:**
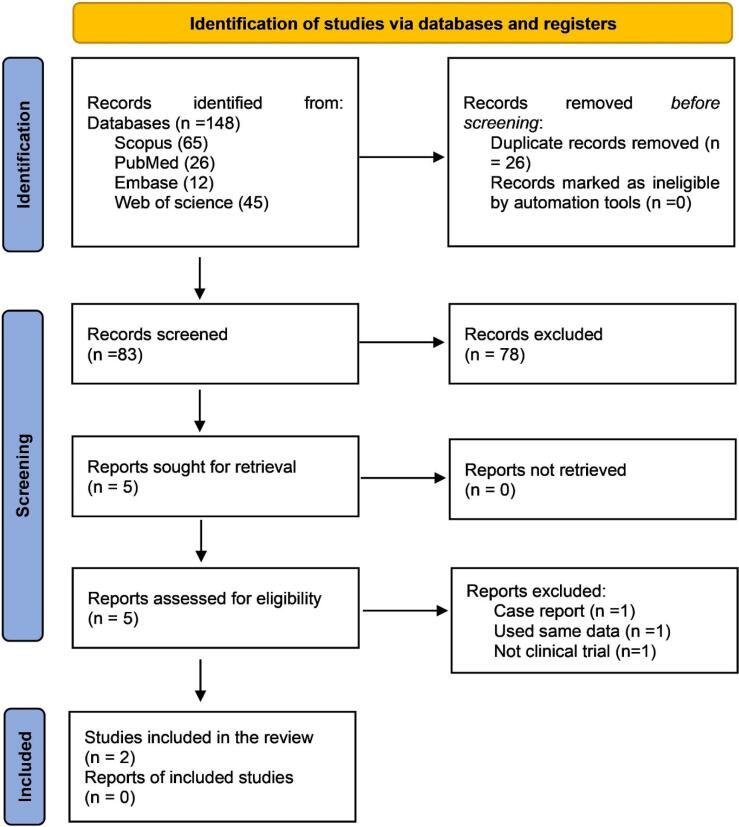
PRISMA flowchart

**Figure 2:**
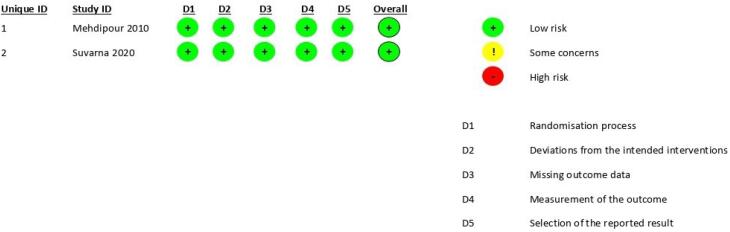
The results of the risk of bias of the studies using the revised Cochrane risk-of-bias tool (RoB2)

 The systematic search resulted in 148 articles. After removing the duplicates, 83 articles remained. A comprehensive assessment of the article’s titles and abstracts was conducted by the review team, and after excluding the unrelated articles to the research objectives, the full texts of five articles were retrieved; one was a case report,^6^ one “25” used the same data as Suvarna et al^31^ and one was not a clinical trial.^32^ Eventually, two articles were included in the study.^15,31^ Figure 1 presents the PRISMA screening flowchart. Based on the revised Cochrane risk-of-bias tool for randomized trials (RoB2) scoring system, both papers had low risks of bias (Figure 2). The population of the study included 60 individuals. Both included articles were randomized clinical trials. Table 1 presents the information obtained from the two articles.

**Table 1:** Data obtained from the included articles

**Author (y)**	**Type of study**	**Country**	**Study group (sample size)**	**Zinc**		**Steroid drugs**		**Pain and Burning (VAS)**		**Lesion size cm**^2^	
				**Form**	**Administration period**	**Form**	**Administration period**	**Baseline**	**After**	**Baseline**	**After**
Mehdipour^12^ 2010	Randomized double blind Clinical Trial	Iran	Control (10)	-	-	fluocinolone ointment	Twice daily for two weeks	3.7	1.6	1.9 (erosive) 2.1 (reticular)	NR
			Case (10)	0.2% mouthwash	Three times daily for two weeks			5.2	1.1	3.1 (erosive) 2.2 (reticular)	NR
Suvarna^31^2020	Single blind clinical Trial	India	Control (20)	-	-	0.1% triamcinolone acetonide	Twice daily for one week	7.6 ± 2.0	0.7 ± 0.6	1.9	0.3
			Case (20)	zinc 50 mg tablets	twice daily for 8 weeks			8.7 ± 1.9	0.6 ± 0.8	1.9	0.25

NR: Not Reported.

## Discussion

 Current treatments for OLP are not curative but are aimed at relieving pain and are, therefore, usually restricted to managing the painful, erosive, and ulcerative forms. Many treatments have been tried, including topical, intra-lesional, and systemic corticosteroids, topical cyclosporine, topical and systemic retinoids, and photochemotherapy.^33^ Approximately 10% of OLP patients present with the disease only localized to the gingiva. Desquamative gingivitis is a clinical description characterized by erythema of the gingiva with desquamation that can progress to ulceration.^34^ Apoptosis occurring due to zinc (an antiapoptotic agent) deficiency is one of the mechanisms involved in the pathogenesis of the lichen planus.^35^ Zinc has antioxidant effects and is involved in cell membrane stabilization; therefore, zinc deficiency can disrupt the elimination of oxidative stress and the destruction of free radicals during inflammation.^36^ Various studies on the relationship between serum zinc levels and the lichen planus indicated the effect of zinc on OLP.^27,37-39^

 The current systematic review investigated the effect of adjuvant zinc on corticosteroid therapy of OLP in clinical trials. The first study included in this study, Mehdipour et al,^15^ investigated the effect of 0.2% zinc mouthwash with fluocinolone ointment on patients with erosive lichen planus. The study revealed that both zinc mouthwash with fluocinolone ointment and the fluocinolone ointment alone effectively reduced pain, burning, and surface areas of the lesions. The reduction in the lesion’s surface area was significantly higher with zinc mouthwash than with the fluocinolone mouthwash alone. However, added zinc did not significantly reduce pain and burning sensation compared with the control group.

 Zinc is essential for epithelial growth, required for normal cell function, and carbohydrate, protein, and lipid metabolism.^39,40^ Infiltration of inflammatory cells, especially CD8, at the epithelium and connective tissue inflammation site, is one of the primary indicators of lichen planus. Zinc can prevent the inflammation process.^41^ Zinc is also an antiapoptotic agent and a cell preserver that protects proteins and nucleic acid from degeneration.^42^

 The second article in the present study was by Suvarna et al,^31^ which investigated the effect of oral zinc and triamcinolone Orabase (0.1%) on improving symptomatic OLP. The study investigated patients aged 30‒70 in two groups receiving 50-mg zinc acetate tablets with 1% triamcinolone and triamcinolone alone. The assessed sites included the buccal mucosa, gingiva, and tongue. The triamcinolone administration continued for one week and was used twice daily; however, zinc tablet intake continued for six months. The six-month follow-up indicated a significant decrease in burning sensation and lesion size in patients with symptomatic OLP in each group, with no significant difference between the groups.

 There are different biological types of zinc for therapeutic applications. Among the forms, zinc sulfate has multiple side effects, including nausea, heartburn, fever, weakness, and fatigue, compared to zinc acetate; therefore, it is rarely used. Suvarna et al^31^ administered zinc acetate to treat OLP patients, and there was no report of adverse effects from the patients during the entire consumption period.

 Zinc activates caspase-3 and causes DNA fragmentation, which leads to keratinocyte apoptosis. Inhibition of matrix metalloproteinase-1 (MMP-1) activation prevents the accumulation of T cells in OLP and prevents MMP-9 from collagen excision. Therefore the integrity of the basement membrane is maintained.^31^

 The effect of zinc on cutaneous lichen planus has been the subject of some research.^43^ Thomas et al^44^ studied the efficacy of combining topical zinc sulfate with clobetasol propionate cream compared to clobetasol propionate cream monotherapy in the treatment of cutaneous lichen planus and reported that the combination was significantly more effective in treating the lesions, especially in the long term.

 A small number of included studies (two) and a search for only English articles are the main limitations of this study. When there are few articles about treating a common disease, the systematic review serves as evidence of absence. Thus, more clinical trials in this field with different doses and forms of zinc are recommended.

## Conclusion

 Based on the findings of the current systematic review, administering zinc, in addition to corticosteroids, did not improve the symptoms compared with corticosteroid monotherapy. Also, the results were inconclusive regarding the effect of added zinc in reducing lesion sizes. Therefore, considering the low number of studies and lack of sufficient evidence, it is not currently possible to reach a definitive conclusion regarding the effectiveness of zinc in treating OLP. Accordingly, further clinical trials are necessary with larger sample sizes on zinc supplementation as an adjunct to corticosteroids for reducing the symptoms of lichen planus and disease improvement.

## Competing Interests

 None.

## Data Availability Statement

 The data are available upon request from the corresponding author.

## Ethical Approval

 This study was approved by the ethical committee of Tabriz University of Medical Sciences (Ethics No. IR.TBZMED.REC.1401.077).

## Funding

 None.
